# Impact of Admission Route on In-Hospital Mortality in Patients with Traumatic Brain Injury: A Retrospective Observational Study of a Single Major Trauma Center in South Korea

**DOI:** 10.3390/jcm15051947

**Published:** 2026-03-04

**Authors:** Jihwan Moon, Sungwook Park

**Affiliations:** Department of Emergency Medicine, Pusan National University Hospital, Busan 49241, Republic of Korea; moon0549@naver.com

**Keywords:** traumatic brain injury, admission route, inter-hospital transfer, mortality, trauma center, clinical outcome

## Abstract

**Background/Objectives:** The optimal transport strategy for patients with traumatic brain injury (TBI) remains debated, particularly in trauma systems where inter-hospital transfer is common. Whether secondary transfer independently influences mortality after risk adjustment is unclear. This study aimed to evaluate the association between admission route and in-hospital mortality among patients with TBI at a major trauma center (MTC). **Methods:** This retrospective observational study included 417 patients with TBI and an Abbreviated Injury Scale (AIS) head score ≥ 3 (direct admission: 245; inter-hospital transfer: 172). Severe TBI was defined as a total Glasgow Coma Scale (GCS) score ≤ 8 or the need for advanced airway management. Multivariable logistic regression was performed to assess whether admission route was independently associated with in-hospital mortality after adjustment for age, physiological status at MTC arrival, and injury severity. Subgroup analysis was conducted in patients with severe TBI. **Results:** Crude mortality was higher in the direct admission group than in the transfer group (40.8% vs. 26.7%; *p* = 0.003), despite significantly longer injury-to-trauma center arrival times in transferred patients (219.0 vs. 44.0 min). In multivariable analysis, admission route was not independently associated with mortality in the overall cohort (adjusted odds ratio [aOR] 0.75; 95% CI 0.44–1.28; *p* = 0.298) or in the severe TBI subgroup (*n* = 233; aOR 0.88; 95% CI 0.47–1.67; *p* = 0.705). Increasing age and lower GCS motor scores were consistently associated with higher mortality in both analyses. **Conclusions:** Inter-hospital transfer was not independently associated with increased in-hospital mortality among patients with TBI. After consideration of patient age and neurological severity, initial stabilization at a nearby hospital followed by transfer may be an acceptable transport strategy for patients who present with physiological instability requiring immediate resuscitative interventions.

## 1. Introduction

Traumatic brain injury (TBI) is a leading cause of mortality and permanent disability among major trauma patients worldwide. The brain is highly sensitive to interruptions in oxygen and blood flow. Beyond the initial mechanical insult—the primary injury—the secondary injury phase, driven by pathophysiological processes such as increased intracranial pressure and hypoxia, further exacerbates clinical outcomes [1]. Unlike irreversible primary injuries, secondary injuries can be minimized through appropriate clinical interventions; thus, preventing them is the cornerstone of TBI management strategies. In this regard, specialized trauma centers play a pivotal role in reducing secondary injury by providing timely and specialized care upon patient arrival [2,3]. Accordingly, many developed countries have established trauma center–based systems to minimize delays in definitive care. However, in South Korea, a substantial number of patients with severe TBI are initially managed at nearby local hospitals and subsequently undergo inter-hospital transfer due to limitations in field triage, regional geographic characteristics, and disparities in trauma care capabilities across regions.

Previous studies investigating outcomes according to admission route have yielded inconsistent results [4,5,6,7,8,9]. A comprehensive systematic review suggested that much of this uncertainty reflects important methodological limitations as well as substantial heterogeneity in trauma system structures, transport distances, and study populations across regions [10]. However, studies from South Korea evaluating the association between admission route to trauma centers and mortality in patients with TBI remain limited. In addition, the scope of prehospital interventions within the Korean emergency medical services (EMS) system differs from that in some other trauma systems. Prehospital providers in Korea operate under defined procedural limitations, which may restrict the use of certain advanced resuscitative interventions during transport. As a result, the clinical implications of direct transport versus inter-hospital transfer may not be directly comparable to those reported in systems where broader prehospital interventions are routinely available.

Therefore, this study aimed to evaluate the association between admission route and in-hospital mortality among patients with TBI. In addition, we assessed whether this association differed among patients with severe TBI. We hypothesized that inter-hospital transfer, which is associated with a longer time from injury to trauma center arrival, would be associated with a higher risk of in-hospital mortality compared with direct admission. This study may contribute data relevant to discussions on prehospital transport strategy in systems where prehospital intervention capabilities are constrained.

## 2. Materials and Methods

### 2.1. Study Design

This study was designed as a retrospective observational analysis of patients with TBI who were treated at a single major trauma center (MTC) between January 2022 and December 2024. The MTC is located in Busan, South Korea, and serves as the designated regional trauma center for the Busan metropolitan area and the surrounding South Gyeongsang Province (Gyeongnam) region. The study protocol was reviewed and approved by the Institutional Review Board (IRB) of our institution (IRB No. 2602-007-159). Given the retrospective nature of the study and the use of de-identified data, the requirement for informed consent was waived. This investigation adhered to the principles of the Declaration of Helsinki and followed the STROBE (Strengthening the Reporting of Observational Studies in Epidemiology) guidelines for observational studies.

### 2.2. Study Population and Group Classification

We systematically screened all trauma patients who visited our trauma center during the study period. Inclusion criteria were as follows: (1) age 18 years or older, (2) presence of TBI confirmed by computed tomography, and (3) an Abbreviated Injury Scale (AIS) score of 3 or higher for the head region. Patients were excluded based on the following criteria: (1) age under 18 years, (2) cardiac arrest upon arrival at the trauma center, (3) arrival more than 24 h after the initial injury [11], and (4) TBI with a head AIS score < 3. After applying these criteria, the final study population was categorized into two groups according to the admission route: the direct admission group (patients who came directly from the scene) and the transfer group (patients referred from local hospitals).

### 2.3. Data Collection

Patient data were systematically retrieved from the institutional electronic medical records (EMR). We collected comprehensive baseline demographic information, including age and sex. To evaluate the prehospital phase, the mechanism of injury and the interval from injury to MTC arrival were recorded. For the transfer group, the time from injury to local hospital arrival was also documented. Clinical status upon arrival at the trauma center was assessed using physiological parameters, including systolic blood pressure (SBP), oxygen saturation (SpO_2_), advanced airway (endotracheal intubation or supraglottic devices), and the total Glasgow Coma Scale (GCS) score and its motor component. When SBP was not measurable on arrival due to profound hypotension, these cases were categorized as “unmeasurable” rather than treated as missing values, as this condition reflects extreme physiologic instability. Similarly, SpO_2_ values that could not be obtained because of severe hypoxia or poor perfusion were classified as “unmeasurable,” representing a clinically significant physiologic state. These variables were analyzed as categorical variables including the “unmeasurable” category. Anatomical injury severity was quantified using the AIS for each body region and the Injury Severity Score (ISS). We also collected data on acute phase interventions, blood transfusion requirements within 4 and 24 h of admission, and whether emergency surgery or transarterial embolization (TAE) was performed. For the purpose of post hoc subgroup analysis, we additionally retrieved data on the performance of neurosurgical procedures, including craniotomy, craniectomy, burr hole insertion, and ICP monitor insertion, from the electronic medical records.

### 2.4. Definition of Severe TBI

Although an AIS head score of 3 or higher indicates clinically significant anatomical brain injury, it does not necessarily reflect immediate physiological or neurological instability. Therefore, severe TBI was defined as a total GCS score ≤ 8, in accordance with established criteria [12]. Patients requiring advanced airway management were also classified as having severe TBI because their total GCS could not be reliably assessed.

Because total GCS may be unreliable or incomplete in patients with advanced airway devices, the GCS motor score was additionally analyzed as a measure of neurological severity. The motor component can be assessed in nearly all patients, including those with advanced airway placement, allowing for more consistent comparison across groups. Prior studies have also demonstrated the strong prognostic value of the GCS motor component in the patients with TBI [13,14].

### 2.5. Outcome

The primary outcome was in-hospital mortality, defined as death from any cause during the index hospitalization.

### 2.6. Statistical Analysis

Continuous variables are presented as medians with interquartile ranges (IQRs). Normality was assessed using the Shapiro–Wilk test, and between-group comparisons were performed using the Mann–Whitney U test. Categorical variables are expressed as numbers with percentages and were analyzed using the chi-square test or Fisher’s exact test, as appropriate.

To evaluate whether the admission route independently affected in-hospital mortality, multivariable logistic regression analysis was performed using a stepwise hierarchical modeling approach, in which covariates were sequentially added to assess their confounding effects on the association between admission route and mortality. The admission route was entered as the primary independent variable. Model 1 was adjusted for age and sex. Model 2 was further adjusted for physiological parameters at trauma center arrival, including SBP category, oxygen saturation category, and the GCS motor score. Model 3, the final model, additionally included ISS and therapeutic interventions (emergency surgery or transarterial embolization).

Multicollinearity among covariates was assessed using variance inflation factors. Model calibration was evaluated with the Hosmer–Lemeshow goodness-of-fit test. Results are presented as adjusted odds ratios (ORs) with 95% confidence intervals (CIs).

Predefined subgroup analysis was conducted among patients with severe TBI, to examine whether the association between admission route and mortality differed according to neurological severity. Additionally, a post hoc subgroup analysis was performed among patients who underwent neurosurgical procedures to further examine the association between admission route and mortality in this specific population. A post hoc power analysis was also performed to assess the statistical adequacy of the sample size. All statistical analyses were performed using MedCalc for Windows, version 23.0.5 (MedCalc Software Ltd., Ostend, Belgium). All tests were two-sided, and a *p*-value < 0.05 was considered statistically significant.

## 3. Results

### 3.1. Baseline Characteristics of the Study Population

**Figure 1:**
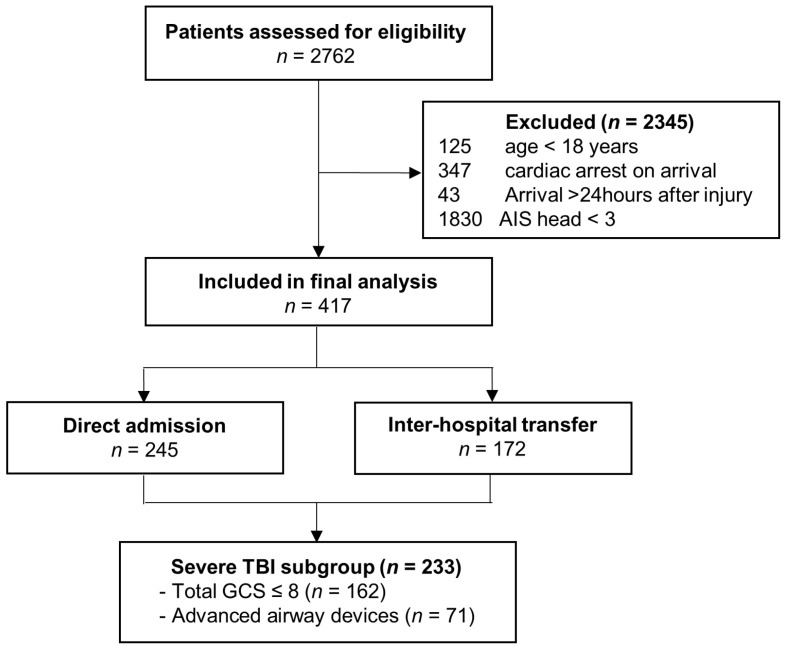
Flow diagram of patient selection and study cohort formation. (AIS, abbreviated Injury Scale; TBI, traumatic brain injury).

During the study period, a total of 2762 trauma patients visited the trauma center. We excluded 125 patients aged under 18 years, 347 patients with cardiac arrest on arrival, and 43 patients who arrived more than 24 h after the injury. Additionally, 1830 patients with AIS head score < 3 were excluded. Consequently, 417 patients met the inclusion criteria and were enrolled in this study, comprising 245 in the direct admission group and 172 in the transfer group (Figure 1, Table 1).

The transfer group was significantly older than the direct admission group (median 66.0 vs. 62.0 years; *p* = 0.016). Although the mechanism of injury varied between the groups (*p* < 0.001), the transfer group experienced a significantly longer median interval from injury to trauma center arrival (219.0 vs. 44.0 min; *p* < 0.001). Upon arrival at the trauma center, the direct admission group presented with more unstable physiological status, including higher rates of unmeasurable SBP (14.7% vs. 1.2%; *p* < 0.001) and oxygen saturation (13.5% vs. 0.6%; *p* < 0.001). In-hospital mortality was significantly higher in the direct admission group compared to the transfer group (40.8% vs. 26.7%; *p* = 0.003). A post hoc power analysis based on the observed mortality rates demonstrated that the study achieved a statistical power of 85.1% (Cohen’s h = 0.030, two-sided α = 0.05), exceeding the conventional 80% threshold.

### 3.2. Comparison Between Survivors and Non-Survivors in the Total Population

When comparing clinical characteristics based on survival in the total study population (Table 2), non-survivors were significantly older (median 68.0 vs. 62.0 years; *p* < 0.001) and were less likely to be transferred from a local hospital (31.5% vs. 46.5%; *p* = 0.003).

At the time of trauma center admission, non-survivors showed a higher frequency of unmeasurable SBP (19.9% vs. 3.3%; *p* < 0.001) and oxygen saturation (19.2% vs. 2.2%; *p* < 0.001). The median GCS motor score was notably lower in non-survivors (2.0 vs. 5.0; *p* < 0.001), while the ISS was significantly higher compared to survivors (25.5 vs. 25.0; *p* < 0.001).

### 3.3. Multivariate Analysis of Mortality in the Total Population

Multivariate logistic regression was performed to identify factors associated with in-hospital mortality in the total population (Table 3). In Model 1, which adjusted for age and sex, the transfer route was significantly associated with decreased mortality (aOR 0.47; 95% CI 0.30–0.72; *p* < 0.001). However, this significance disappeared in Model 2 after adjusting for physiological parameters and GCS motor score (aOR 0.75; 95% CI 0.44–1.27; *p* = 0.290). In the final model (Model 3), the admission route remained non-significant (*p* = 0.298), while age, unmeasurable oxygen saturation, GCS motor score, and ISS were confirmed as independent predictors of mortality.

In all models, admission route was included as the primary independent variable.

Model 1 was adjusted for age and sex.

Model 2 was further adjusted for admission systolic blood pressure, saturation and GCS motor score.

Model 3 was further adjusted for Injury Severity Score and surgery/TAE status.

### 3.4. Subgroup Analysis of Patients with Severe TBI

A subgroup analysis was conducted on 233 patients with severe TBI (Table 4).

In this specific cohort, the proportion of transferred patients did not differ significantly between survivors and non-survivors (38.9% vs. 31.2%; *p* = 0.220). Non-survivors were older (median 66.0 vs. 56.5 years; *p* < 0.001) and had higher rates of unmeasurable SBP (22.4% vs. 7.4%; *p* = 0.007) and oxygen saturation (21.6% vs. 5.6%; *p* < 0.001). The GCS motor score was significantly lower in the mortality group compared to the survival group (median 1.0 vs. 3.0; *p* < 0.001).

### 3.5. Multivariate Analysis in the Severe TBI Subgroup

In the multivariate analysis restricted to the severe TBI subgroup (Table 5), the admission route was not significantly associated with mortality across all models. In the final adjusted model (Model 3), the aOR for the transfer group was 0.88 (95% CI 0.47–1.67; *p* = 0.705). Age (aOR 1.05; 95% CI 1.03–1.07; *p* < 0.001) and GCS motor score (aOR 0.67; 95% CI 0.53–0.84; *p* < 0.001) remained independent predictors, whereas ISS did not show statistical significance in this subgroup (*p* = 0.435).

In all models, admission route was included as the primary independent variable.

Model 1 was adjusted for age and sex.

Model 2 was further adjusted for admission systolic blood pressure, saturation and GCS motor score.

Model 3 was further adjusted for Injury Severity Score and surgery/TAE status.

### 3.6. Post Hoc Subgroup Analysis of Patients Undergoing Neurosurgical Procedures

Among the total study patients of 417 patients, 102 patients underwent neurosurgical procedures. The proportion of patients undergoing neurosurgical procedures did not differ significantly between the direct admission and transfer groups (64/245 [26.1%] vs. 38/172 [22.1%]; *p* = 0.346). In this subgroup, multivariable logistic regression analysis adjusting for age, GCS motor score, and ISS demonstrated that admission route was not independently associated with in-hospital mortality (aOR 0.98; 95% CI 0.38–2.54; *p* = 0.970). Age (aOR 1.05; 95% CI 1.02–1.08; *p* = 0.002), GCS motor score (aOR 0.67; 95% CI 0.51–0.88; *p* = 0.004), and ISS (aOR 1.07; 95% CI 1.00–1.14; *p* = 0.036) remained independent predictors of in-hospital mortality in this subgroup.

## 4. Discussion

This retrospective observational study investigated whether the admission route serves as an independent predictor of in-hospital mortality among patients with TBI at an MTC. Our analysis revealed that the admission route—direct admission from the scene versus inter-hospital transfer—was not independently associated with in-hospital mortality after adjusting for age, physiological status, and injury severity. This finding remained consistent in the subgroup analysis of patients with severe TBI.

Our findings are consistent with large multicenter studies showing no independent mortality effect of inter-hospital transfer when appropriate risk adjustment is performed. The European CENTER-TBI study (1347 patients from 53 centers) found no association between early secondary referral and functional outcome (OR 1.07, 95% CI 0.78–1.69) or survival (OR 1.05, 95% CI 0.58–1.90) [15]. Similarly, Sy et al. [16] reported no impact of transportation time on mortality (OR 0.98, 95% CI 0.95–1.01) among 2860 severe TBI patients in British Columbia, and Gale et al. [17] found that neither transfer distance nor time predicted mortality in rural trauma. These studies support that early physiological stabilization may be as important as transport-related factors, including admission route, transport time, and distance, in influencing outcomes. However, some studies have reported different outcomes between two groups. Joosse et al. [18] found higher mortality in transferred patients requiring emergency neurosurgical intervention within 6 h (33% vs. 27%, *p* = 0.553), though this difference did not reach statistical significance. The Hasler et al. [19] Swiss registry study reported elevated mortality among direct admissions (HR 1.63, 95% CI 1.40–1.89), likely reflecting more severe baseline physiological instability—similar to the higher unmeasurable vital signs rates in our direct admission group. Two systematic reviews synthesize the broader evidence. Pickering et al. [10] found no mortality difference for initial triage to non-specialist versus specialist centers (OR 1.03, 95% CI 0.85–1.23). The more recent Jones et al. [20] review noted that while direct transport showed some benefits, secondary transfers were actually associated with reduced 24 h (RR 0.31) and 30-day mortality (RR 0.66) in certain studies.

Taken together, our findings, interpreted alongside the existing literature, suggest that decisions regarding direct transport to a trauma center versus initial stabilization at a local hospital should be guided by patient-specific risk profiles rather than a uniform time-based strategy. In our study, age and neurological status, as reflected by the GCS motor score, were the most consistent independent predictors of mortality across both the overall cohort and the severe TBI subgroup. These findings suggest that older patients presenting with depressed motor responses represent a particularly high-risk group in whom early access to definitive trauma and neurosurgical care may be most critical. Indeed, older adults with TBI have been shown to be disproportionately vulnerable to worse clinical outcomes and increased mortality, partly due to age-related reductions in physiological reserve and higher prevalence of comorbidities, even after relatively mild mechanisms of injury [21,22]. For such patients, direct transport to an MTC may be preferentially considered when feasible. This risk-based perspective aligns with prior evidence emphasizing the prognostic importance of age and neurological status in TBI. In contrast, patients who are relatively younger and who do not demonstrate profound neurological impairment may derive less benefit from bypassing nearby facilities, particularly when severe physiological derangements require immediate correction. Our observation that transferred patients arrived with fewer unmeasurable vital signs supports the possibility that early physiological stabilization at referring hospitals may mitigate the adverse impact of transfer-related delays in selected cases. Thus, for patients without extreme neurological depression, prioritizing airway protection, oxygenation, and hemodynamic stabilization before transfer may represent a safe and rational strategy within integrated trauma systems. Importantly, these interpretations should not be understood as endorsing routine delays in definitive care, but rather as highlighting that physiological optimization and patient risk stratification are central to triage decisions in suspected TBI. Trauma system performance should therefore be evaluated not solely by transport time metrics, but also by the ability to identify high-risk neurological presentations in the field and to deliver effective early resuscitation when immediate transfer is not feasible.

A key methodological strength of our study is the use of hierarchical regression with sequential adjustment for progressively detailed confounders to address potential transfer survival bias. This approach allowed us to systematically disentangle the effects of transfer from baseline patient characteristics by controlling for demographics in Model 1, adding physiological parameters at trauma center arrival in Model 2, and further incorporating injury severity and treatment factors in Model 3. Furthermore, our separate analysis of the suspected severe TBI subgroup strengthened the validity of findings across different injury severity strata.

## 5. Study Limitations

This study has several limitations. First, as a retrospective analysis conducted at a single trauma center, the findings may not be generalizable to regions with different trauma system structures. As an observational study, causal inferences cannot be made. Second, we lacked information on patients evaluated at referring hospitals who were not transferred, including the reasons for non-transfer. This may have introduced selection bias, as transferred patients may represent a selectively triaged or stabilized subgroup rather than the full spectrum of injury severity within the referral population. Third, physiological data at the time of initial EMS contact were available for directly admitted patients but not for transferred patients. Consequently, baseline physiological status at a comparable prehospital time point could not be directly contrasted between groups. Because transferred patients were assessed at the trauma center after initial management at referring hospitals, residual differences in early injury severity may have persisted despite adjustment for arrival physiology. Fourth, detailed information on clinical interventions performed at referring to hospitals prior to transfer was unavailable. The absence of data on fluid resuscitation, blood transfusion, and vasopressor use limited assessment of the quality of early stabilization. Fifth, prehospital care data from EMS, including field triage practices and treatments, were not available, and variations in these factors may have influenced patients’ physiological condition upon arrival. Sixth, classification of “unrecordable” vital signs in the most critically ill patients may have resulted in some misclassification, although these cases were treated as a distinct physiological category. Seventh, pupillary reactivity data were not incorporated into our neurological severity assessment. Formal pupillary examination was frequently deferred during initial resuscitation in hemodynamically unstable patients, and when recorded, assessments were often performed several minutes after arrival, potentially failing to accurately reflect the initial neurological status. Therefore, the use of the GCS Pupils score was not feasible, which may have limited the precision of neurological severity adjustment. Finally, functional outcomes beyond in-hospital mortality were not evaluated, restricting assessment of the broader impact of admission route on long-term neurological recovery. Despite multivariable adjustment, residual confounding from unmeasured factors may persist.

## 6. Conclusions

In conclusion, admission route, including inter-hospital transfer, was not independently associated with in-hospital mortality among patients with TBI after adjustment for injury severity and physiological status. Despite substantially longer times to trauma center arrival, transferred patients had outcomes comparable to those directly admitted. These findings suggest that transport strategy decisions in suspected TBI should not be based solely on minimizing time to trauma center arrival, but rather on patient condition and the need for immediate physiological stabilization.

## Figures and Tables

**Table 1:** Comparison of baseline characteristics between direct admission and transferred patients.

	Direct (n = 245)	Transfer (n = 172)	*p*-Value
Age, years	62.0 (45.0–72.0)	66.0 (54.0–76.5)	0.016
Males, *n* (%)	174 (71.0)	119 (69.2)	0.687
Mechanism of injury, *n* (%)			<0.001
Traffic accident	113 (46.1)	66 (38.4)	
Fall from height	94 (38.4)	56 (32.6)	
Ground-level fall	15 (6.1)	36 (20.9)	
Struck by object	10 (4.1)	10 (5.8)	
Cutting or piercing	1 (0.4)	0 (0.0)	
Others	12 (4.9)	4 (2.3)	
Time from injury to MTC arrival (min)	44.0 (32.0–60.0)	219.0 (154.5–344.5)	<0.001
Time from injury to LH arrival (min)	-	65.5 (33.0–540.0)	
MTC admission vital signs			
Systolic blood pressure, *n* (%)			<0.001
≥90 mmHg	162 (66.1)	150 (87.2)	
<90 mmHg	47 (19.2)	20 (11.6)	
Unmeasurable	36 (14.7)	2 (1.2)	
Saturation, *n* (%)			<0.001
≥90%	194 (79.2)	168 (97.7)	
<90%	18 (7.3)	3 (1.7)	
Unmeasurable	33 (13.5)	1 (0.6)	
Glasgow Coma Scale			
Motor score	4.0 (1.0–5.0)	5.0 (3.0–6.0)	<0.001
Advance airway on arrival	19 (7.8)	52 (30.2)	<0.001
AIS ≥ 3 extra-cranial injuries			
Face	0 (0.0)	0 (0.0)	-
Thorax	62 (25.3)	41 (23.8)	0.732
Abdomen/Pelvic contents	20 (8.2)	7 (4.1)	0.095
Spine/extremities	19 (7.8)	9 (5.2)	0.312
Outcomes of trauma			
Injury Severity Score	25.0 (22.0–33.0)	25.0 (22.0–29.0)	0.120
4 h PRBC ≥ 3 units, *n* (%)	89 (36.3)	32 (18.6)	<0.001
24 h PRBC ≥ 10 units, *n* (%)	13 (5.3)	2 (1.2)	0.026
Surgery or TAE, *n* (%)	97 (39.6)	67 (39.0)	0.896
Neurosurgical procedures, *n* (%)	64 (26.1)	38 (22.1)	0.346
In-hospital mortality, *n* (%)	100 (40.8)	46 (26.7)	0.003

MTC, major trauma center; LH, local hospital; AIS, abbreviated injury scale; PRBC, Packed red blood cell; TAE, transarterial embolization. Neurosurgical procedures include craniotomy, craniectomy, burr hole insertion and intracranial pressure monitor insertion. Values are presented as median (interquartile range) or number (%). Data were analyzed using the Mann–Whitney U test, Chi-square test, or Fisher’s exact test as appropriate.

**Table 2:** Comparison of baseline characteristics between survivors and non-survivors in the total study population.

	Survival (n = 271)	Mortality (n = 146)	*p*-Value
Age, years	62.0 (44.0–71.0)	68.0 (54.0–81.0)	<0.001
Males, *n* (%)	191 (70.5)	102 (69.9)	0.896
Mechanism of injury, *n* (%)			0.560
Traffic accident	119 (43.9)	60 (41.1)	
Fall from height	96 (35.4)	54 (37.0)	
Ground-level fall	30 (11.1)	21 (14.4)	
Struck by object	15 (5.5)	5 (3.4)	
Cutting or piercing	0 (0.0)	1 (0.7)	
Others	11 (4.1)	5 (3.4)	
Admission route, *n* (%)			0.003
Transfer from LH	126 (46.5)	46 (31.5)	
Time from injury to MTC arrival (min)	91.0 (44.0–227.8)	56.0 (34.0–154.0)	<0.001
Time from injury to LH arrival (min)	63.0 (33.0–551.0)	81.0 (30.0–379.0)	0.901
MTC admission vital signs			
Systolic blood pressure, *n* (%)			<0.001
≥90 mmHg	215 (79.3)	97 (66.4)	
<90 mmHg	47 (17.3)	20 (13.7)	
Unmeasurable	9 (3.3)	29 (19.9)	
Saturation, *n* (%)			<0.001
≥90%	257 (94.8)	105 (71.9)	
<90%	8 (3.0)	13 (8.9)	
Unmeasurable	6 (2.2)	28 (19.2)	
Glasgow Coma Scale			
Motor score	5.0 (4.0–6.0)	2.0 (1.0–4.0)	<0.001
Advance airway on arrival	30 (11.1)	41 (28.1)	<0.001
AIS ≥3 extra-cranial injuries			
Face	0 (0.0)	0 (0.0)	-
Thorax	66 (24.4)	37 (25.3)	0.824
Abdomen/Pelvic contents	18 (6.6)	9 (6.2)	0.850
Spine/extremities	16 (5.9)	12 (8.2)	0.810
Outcomes of trauma			
Injury Severity Score	25.0 (18.3–29.0)	25.5 (25.0–34.0)	<0.001
4 h PRBC ≥ 3 units, *n* (%)	57 (21.0)	64 (43.8)	<0.001
24 h PRBC ≥ 10 units, *n* (%)	5 (1.8)	10 (6.8)	0.009
Surgery or TAE, *n* (%)	99 (36.5)	65 (44.5)	0.112
Neurosurgical procedures, *n* (%)	61 (22.5)	41 (28.1)	0.207

**Table 3:** Multivariate logistic regression analysis of factors associated with in-hospital mortality in the total study population.

	Model 1		Model 2		Model 3	
Variable	aOR (CI)	*p*-Value	aOR (CI)	*p*-Value	aOR (CI)	*p*-Value
Age	1.02 (1.01–1.03)	<0.001	1.05 (1.03–1.07)	<0.001	1.05 (1.03–1.07)	<0.001
Sex		0.538		0.333		0.285
Male	reference		reference		reference	
Female	0.86 (0.54–1.37)		0.76 (0.44–1.32)		0.74 (0.42–1.29)	
Admission route		<0.001		0.290		0.298
Direct	reference		reference		reference	
Transfer	0.47 (0.30–0.72)		0.75 (0.44–1.27)		0.75 (0.44–1.28)	
Admission SBP						
≥90 mmHg			reference		reference	
<90 mmHg			0.81 (0.39–1.68)	0.563	0.67 (0.31–1.44)	0.300
Unmeasurable			1.21 (0.31–4.68)	0.785	0.80 (0.19–3.36)	0.760
Admission saturation						
≥90%			reference		reference	
<90%			3.20 (1.02–10.03)	0.046	3.40 (1.05–10.98)	0.041
Unmeasurable			4.54 (1.17–17.63)	0.029	5.60 (1.37–22.86)	0.016
Admission GCS motor			0.54 (0.46–0.62)	<0.001	0.54 (0.46–0.62)	<0.001
Injury Severity Score					1.03 (1.01–1.06)	0.028
Surgery/TAE						0.763
Yes					reference	
No					0.92 (0.53–1.59)	

aOR, adjusted odds ratio: CI, confidence interval; SBP, systolic blood pressure; GCS, Glasgow Coma Scale; TAE, transarterial embolization.

**Table 4:** Comparison of baseline characteristics between survivors and non-survivors among patient with suspected severe TBI (GCS ≤ 8 or advanced airway on arrival).

	Survival (n = 108)	Mortality (n = 125)	*p*-Value
Age, years	56.5 (39.5–68.0)	66.0 (51.8–76.3)	<0.001
Males, *n* (%)	79 (73.1)	85 (68.0)	0.392
Mechanism of injury, *n* (%)			0.560
Traffic accident	47 (43.5)	52 (41.6)	
Fall from height	35 (32.4)	47 (37.6)	
Ground level fall	15 (13.9)	15 (12.0)	
Struck by object	5 (4.6)	5 (4.0)	
Cutting or piercing	0 (0.0)	1 (0.8)	
Others	6 (5.6)	5 (4.0)	
Admission route, *n* (%)			0.220
Transfer from LH	42 (38.9)	39 (31.2)	
Time from injury to MTC arrival, (min)	66.0 (39.0–188.5)	55.0 (32.0–151.0)	0.120
Time from injury to LH arrival, (min)	58.0 (29.0–566.0)	55.0 (23.0–298.0)	0.666
MTC admission vital signs			
Systolic blood pressure, *n* (%)			0.007
≥90 mmHg	81 (75.0)	79 (63.2)	
<90 mmHg	19 (17.6)	18 (14.4)	
Unmeasurable	8 (7.4)	28 (22.4)	
Saturation, *n* (%)			<0.001
≥90%	95 (88.0)	86 (68.8)	
<90%	7 (6.5)	12 (9.6)	
Unmeasurable	6 (5.6)	27 (21.6)	
Glasgow Coma Scale			
Motor score	3.0 (2.0–4.0)	1.0 (1.0–3.0)	<0.001
Advance airway on arrival	30 (27.8)	41 (32.8)	0.407
AIS ≥ 3 extra-cranial injuries			
Face	0 (0.0)	0 (0.0)	-
Thorax	25 (23.1)	31 (24.8)	0.769
Abdomen/Pelvic contents	9 (8.3)	7 (5.6)	0.412
Spine/extremities	6 (5.6)	11 (8.8)	0.343
Outcomes of trauma			
Injury Severity Score	26.0 (22.0–30.0)	26.0 (25.0–34.0)	<0.001
4 h PRBC ≥ 3 units, *n* (%)	36 (33.3)	58 (46.4)	0.043
24 h PRBC ≥ 10 units, *n* (%)	2 (1.9)	9 (7.2)	0.056
Surgery or TAE, *n* (%)	64 (59.3)	60 (48.0)	0.087
Neurosurgical procedures, *n* (%)	44 (40.7)	39 (31.2)	0.130

**Table 5:** Multivariate logistic regression analysis of factors associated with in-hospital mortality among patients with suspected severe TBI (GCS ≤ 8 or advanced airway on arrival).

	Model 1		Model 2		Model 3	
Variable	aOR (CI)	*p*-Value	aOR (CI)	*p*-Value	aOR (CI)	*p*-Value
Age	1.03 (1.02–1.05)	<0.001	1.05 (1.03–1.07)	<0.001	1.05 (1.03–1.07)	<0.001
Sex		0.845		0.996		0.927
Male	reference		reference		reference	
Female	1.06 (0.58–1.93)		1.00 (0.52–1.92)		0.97 (0.50–1.87)	
Admission route		0.073		0.647		0.705
Direct	reference		reference		reference	
Transfer	0.59 (0.34–1.05)		0.86 (0.46–1.62)		0.88 (0.47–1.67)	
Admission SBP						
≥90 mmHg			reference		reference	
<90 mmHg			0.95 (0.41–2.20)	0.902	0.85 (0.35–2.05)	0.721
Unmeasurable			1.43 (0.33–6.32)	0.633	1.17 (0.25–5.52)	0.840
Admission saturation						
≥90%			reference		reference	
<90%			2.52 (0.77–8.26)	0.128	2.78 (0.84–9.26)	0.095
Unmeasurable			3.80 (0.89–16.19)	0.071	4.09 (0.93–17.91)	0.062
Admission GCS motor			0.66 (0.53–0.83)	<0.001	0.67 (0.53–0.84)	<0.001
Injury Severity Score					1.01 (0.98–1.05)	0.435
Surgery/TAE						0.355
Yes					reference	
No					1.34 (0.72–2.48)	

## Data Availability

The datasets generated and/or analyzed during the current study are not publicly available due to hospital policy but are available from the corresponding author on reasonable request.
